# Human-Centric Lighting: Rare-Earth-Free Photoluminescent Materials for Correlated Color Temperature Tunable White LEDs

**DOI:** 10.3390/ijms24043602

**Published:** 2023-02-10

**Authors:** Amador Menéndez-Velázquez, Ana Belén García-Delgado, Dolores Morales

**Affiliations:** Photoactive Materials Research Unit, IDONIAL Technology Center, 33417 Avilés, Asturias, Spain

**Keywords:** human-centric lighting, circadian rhythms, tunable spectrum of WLEDs, spectral conversion, rare-earth-free materials, photoluminescent organic materials

## Abstract

Artificial lighting is ubiquitous in modern society, with detrimental effects on sleep and health. The reason for this is that light is responsible not only for vision but also for non-visual functions, such as the regulation of the circadian system. To avoid circadian disruption, artificial lighting should be dynamic, changing throughout the day in a manner comparable to natural light in terms of both light intensity and associated color temperature. This is one of the main goals of human-centric lighting. Regarding the type of materials, the majority of white light-emitting diodes (WLEDs) make use of rare-earth photoluminescent materials; therefore, WLED development is at serious risk due to the explosive growth in demand for these materials and a monopoly on sources of supply. Photoluminescent organic compounds are a considerable and promising alternative. In this article, we present several WLEDs that were manufactured using a blue LED chip as the excitation source and two photoluminescent organic dyes (Coumarin 6 and Nile Red) embedded in flexible layers, which function as spectral converters in a multilayer remote phosphor arrangement. The correlated color temperature (CCT) values range from 2975 K to 6261 K, while light quality is preserved with chromatic reproduction index (CRI) values superior to 80. Our findings illustrate for the first time the enormous potential of organic materials for supporting human-centric lighting.

## 1. Introduction

Life on planet Earth started around 3.8 billion years ago. Considering that Earth was created approximately 4.6 billion years ago, life has been linked to our planet almost since its inception. A constant factor throughout the history of life has been the constant rotation of the Earth, which produces cycles of light and darkness; in short, the cycles of day and night. Life on Earth evolved under these light patterns [1].

### 1.1. Our Modern Relationship with Light

Our lifestyle and relationship with light has changed dramatically in just a few generations. This transformation is largely attributable to the second industrial revolution, which brought numerous technological innovations, including electricity. Electricity has enabled the creation of artificial light, along with all of its benefits. Artificial light has made it possible to extend the length of days; however, it has also meant “the end of the night”, which has detrimental biological effects on different living beings [2,3,4,5,6], including humans [7,8].

Over the course of the past two centuries, human activities have produced a fundamental shift in society, initiating considerable amounts of migration from the countryside toward an urban existence. Consequently, human beings have moved from a primarily outdoors life to one located indoors, restricting the availability of natural light and increasing our exposure to artificial light.

### 1.2. Circadian Rhythms, Light and Health

Regarding intensity and spectrum characteristics, natural and artificial light are quite different from one another. In addition, we are also exposed to artificial light at inappropriate hours. These facts alter our circadian rhythms, which are 24 h biological rhythms, resulting in a condition referred to as chronodisruption or circadian disruption, with detrimental consequences for our health [9]. An increasing amount of scientific and clinical data revealed that chronodisruption is linked to a variety of illnesses [10], including cancer [11], cardiovascular diseases, and neurodegenerative disorders.

The human circadian system is synchronized with external factors, mainly through the variations in sunlight throughout the day. In the morning, sunlight has a high level of blue radiation, inhibiting the secretion of melatonin. Over the course of the day, the fraction of blue decreases until it is almost null at dusk. Without being exposed to blue light, our body secretes melatonin, a hormone that helps us to fall asleep and which is the main marker of the circadian system.

Melatonin has other health-promoting qualities in addition to being a key sleep regulator. It is a potent anti-inflammatory and antioxidant molecule that also boosts our immune system [12]. Thus, melatonin is essential in the fight against illnesses, such as COVID-19, whose detrimental effects on people can be mitigated by a robust immune system [13].

The identification of a novel class of photoreceptors on the retina at the turn of the century considerably advanced research into circadian rhythms [14]. These photoreceptors contain the photosensitive melanopsin protein and are called intrinsically photosensitive retinal ganglion cells (ipRGCs). Contrary to rods and cones, ipRCGs cells are not involved in the vision process. Using their own photosensitive abilities and data gathered from other photoreceptors (rods and cones), they impact the regulation of the hormone melatonin and, by extension, the human circadian clock.

Melanopsin is a type of photopigment that is especially sensitive to the blue part of the visible spectrum because of its high sensitivity to shorter wavelengths of light (peaking at 480 nm) [15,16]. However, due to the contributions of rods and cones, the spectral sensitivity of the circadian machinery does not completely match that of melanopsin. Methods for the accurate prediction of circadian system reactions to different wavelengths require further development. Results from different experiments based on the human retina’s exposure to different wavelengths of light suggest that the spectral sensitivity of the human circadian system peaks around 460 nm, having a full-width half maximum of 100 nm [17,18].

Following the natural cycle of daylight and darkness is the best way to avoid chronodisruption and its related diseases. Therefore, it is necessary to develop dynamic luminaries that mimic the solar cycle and its variations throughout the day, both in terms of intensity and spectral distribution, paying special attention to the short wavelengths that most interfere with our circadian system [19,20]. In short, we must converge technological and biological evolution to preserve our health. The lighting industry must not only consider energy efficiency issues [21] but also lighting effects on human beings [22,23]. Lighting designed with the needs of people in mind takes both the visual and non-visual effects of light into account and is referred as human-centric lighting. It has become a trending issue among lighting designers and scientists around the world.

### 1.3. Rare-Earth-Free Tunable White LEDs

White light-emitting diodes (WLEDs) have garnered considerable attention due to their notable characteristics, such as high efficiency, small size, and extended operating lifetime. Additionally, unlike other luminaires, such as incandescent bulbs, the spectral distribution of the light and correlated color temperature (CCT) generated by these WLEDs can be tuned and adjusted to specific requirements. Therefore, WLED luminaires that mimic sunlight at different times of the day can be developed.

The vast majority of commercially available WLEDs are made from a blue LED chip and phosphors (photoluminescent wavelength converters). A portion of the blue photons emitted by the chip is absorbed by the phosphors and spectrally converted to lower-energy photons. The combination of blue and down-converted photons appears white. Therefore, commercial WLEDs belong to the category of phosphor-converted WLEDs (pc-WLEDs). This technology was made possible by the successful development of efficient gallium nitride (GaN)-based blue LED chips by Shuji Nakamura, Hiroshi Amano, and Isamu Akasaki, who earned the Nobel Prize in Physics in 2014 for their achievements [24].

The spectrum of light emitted by pc-WLEDs can be tuned by modifying the excitation light source and/or the phosphors (spectral converters). Currently, the most popular phosphors on the market are made from rare-earth elements (REEs) [25]. However, because the vast majority (more than 95%) of worldwide rare-earth elements production is concentrated in a small number of geographical regions (especially in China), there are a number of disadvantages and potential risks to the REE supply chain [26]. There is a critical need for reliable REE substitutes in photonic devices, including lighting systems. Organic materials are promising substitutes.

Solution processability, cheap cost, and low toxicity are a few of the reasons why photoluminescent organic materials [27] are attractive as wavelength converters for photonic technologies, with applications across a variety of industries, including photovoltaics [28,29,30], agriculture [31,32], ophthalmology [33], etc. In addition, the use of photoluminescent organic materials can also be advantageous for LED lighting [34,35,36,37].

Photoluminescent quantum dots are another possible alternative to REEs. They have found application in flat panel displays due to their narrow emission that yields clean colors [38]. However, broader emission is achieved with photoluminescent organic materials. This optical property makes them more useful for ambient lighting applications such as those pursued in this article, in which the emitted light must cover the full visible spectrum in order to achieve good quality lighting with high chromatic reproduction index (CRI). With respect to the absorption, quantum dots have a broader absorption spectrum compared to organic dyes. However, if the organic dye’s absorption spectrum overlaps with the LED chip’s emission range, this will not be an issue. When environmental aspects are considered, organic dyes provide another added advantage over quantum dots because the most efficient photoluminescent quantum dots make use of a very toxic metal such as Cd. In contrast, most organic dyes are environmentally friendly.

We used organic materials as a replacement for rare-earth materials. By doping the ethylene vinyl acetate (EVA) copolymer with two photoluminescent organic dyes (Coumarin 6 and Nile Red) and simply changing the concentration of the photoluminescent species and/or the thickness and/or relative position of the photoluminescent layers, we generated light of different color temperatures ranging from 2975 K to 6261 K. At the same time, we preserved WLED light quality, which is measured through the CRI parameter and takes values greater than 80. Our achievements show the potential of these organic materials in the field of human-centric lighting. To the best of our knowledge, this is the first time that a huge range of CCT values with such high CRI values is achieved with WLEDs by making use of just two photoluminescent dyes working as spectral converters in a flexible remote configuration.

## 2. Results and Discussion

Blue LED chips made of GaN-based materials and some sort of phosphor, either directly applied to the chips themselves or kept apart from the them (remote phosphor arrangement) [39,40], are used in the majority of white LEDs currently on the market. The excitation source and phosphors employed in the production of these WLEDs determine the spectral characteristics of the emitted light. Thus, compared with traditional lighting technologies, WLEDs offer a far higher degree of freedom to choose the light output spectrum. This freedom opens new possibilities for the emission of light with different correlated color temperatures. Here, we report the design of 21 WLEDs. They are built around a blue LED chip and a set of remote organic phosphors.

### 2.1. Design of WLEDs

**Figure 1:**
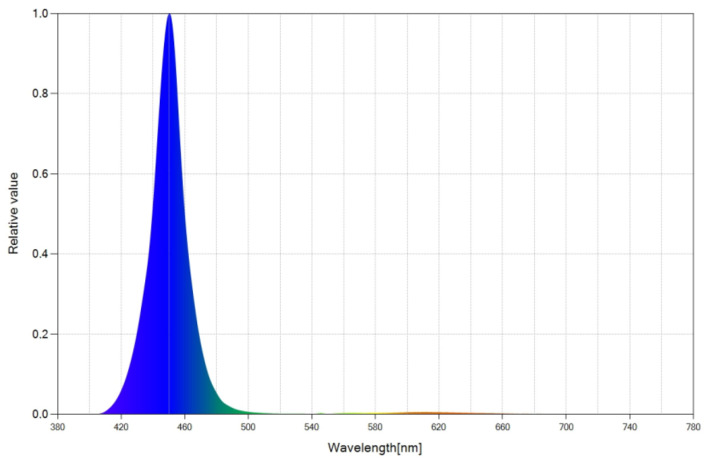
Spectral power distribution (peaking at 450 nm) of selected blue LED chip.

We achieved the excitation of phosphors via a LED chip, whose spectral power distribution (SPD) graph is shown in Figure 1. This LED emits light in the blue region of the spectrum, ranging from 410 nm to 500 nm, reaching a peak at 450 nm.

We used EVA, an optically transparent polymer, to host the photoluminescent organic dyes (Coumarin 6 and Nile Red). The resulting flexible dye-doped EVA layers work as photoluminescent filters (spectral converters) that shift blue light into longer wavelengths, broadening the emitted light’s spectrum in relation to the blue LED chip’s output light. We incorporated each organic dye into a separate layer. It is possible to achieve various spectral conversions by utilizing Coumarin 6-doped layers and/or Nile Red-doped layers and altering their concentrations, thicknesses, and/or relative positions with respect to the blue LED chip. The different concentrations are represented by weight percentages relative to the host material (EVA).

Starting with the mentioned LED chip (see Figure 1) and making use of the 21 different spectral converter configurations, we manufactured 21 different WLEDs. The various spectral converters were divided into two groups: monolayer molecular systems containing just one photoluminescent dye (Section 2.3) and multilayer (two layers) molecular systems containing two distinct photoluminescent dyes (Section 2.4), with one in each layer.

By analyzing the light emitted from the WLEDs, we can determine the optimal configurations for achieving WLEDs with high light quality for different correlated color temperatures. In Section 2.2, we outline and explain the optical characterizations performed on the various WLEDs.

### 2.2. Optical Characterization

The WLED optimization process takes SPD, CRI, and CCT parameters into account.

#### 2.2.1. Spectral Power Distribution (SPD)

The relative power radiated from a light source is plotted against its wavelength in a spectral power distribution (SPD) graph, providing a visual representation of the spectrum of the emitted light. Other parameters, such as CRI and CCT, can be calculated from SPD.

#### 2.2.2. Color Rendering Index (CRI)

The Commission Internationale de l’Éclairage (CIE; International Commission on Illumination) defines the color rendering index (CRI), also referred to as chromatic reproduction index (CRI), as a numerical indicator of a light source’s capacity to faithfully replicate the color of an illuminated object with relation to a reference light source, such as sunlight. The arithmetical mean (R_a_) of the standard CRI points, R_1_–R_8_, based on the CIE 1974 test color samples (TCS), is typically used to report a device’s ability to reproduce colors properly. In the lighting sector, CRI and R_a_ are used interchangeably.

#### 2.2.3. Correlated Color Temperature (CCT)

Correlated color temperature (CCT), which is measured in kelvin (K), describes the color of a light source by contrasting it with the color of a “theoretically perfect radiant” blackbody (an object whose light emission is solely caused by its temperature). The blackbody, similar to any other incandescent body, changes its color as its temperature does.

### 2.3. Monolayer Luminescent Molecular Systems

Using our chosen blue LED chip (see Figure 1) and the photoluminescent organic dye Coumarin 6 (C6) integrated in a single layer, we developed different WLEDs by varying dye concentrations and/or luminescent layer thicknesses. Table 1 lists the top WLEDs created using this approach.

**Figure 2:**
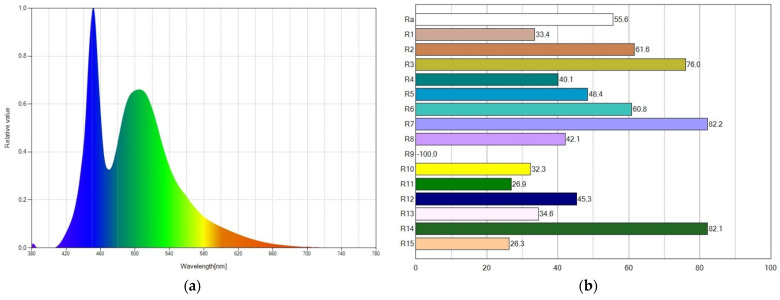
For WLED1, (**a**) spectral power distribution showing a high blue peak and (**b**) CRI values reaching a CRI (R_a_) value of 55.6.

The best results were achieved with WLED 1, providing a CRI of 55.6, which is still very small. The spectral distribution power (SPD) of WLED1 is displayed in Figure 2a. Two peaks from the blue LED chip and Coumarin 6 luminescence are visible in the graph. The associated CRI points are displayed in Figure 2b.

### 2.4. Multilayer Luminescent Molecular Systems

Using the same blue LED chip (see Figure 1) and the photoluminescent organic dyes Coumarin 6 (C6) and Nile Red (NRed) integrated in separate layers, we developed 18 different WLEDs by varying the dye concentrations and/or luminescent layer thicknesses and/or relative positions of the luminescent layers with respect to the blue LED chip. In each of these WLEDs, Layer 1 refers to the layer that is closest to the blue LED chip, and Layer 2 refers to the layer that is farthest from it. By using two dyes (Coumarin 6 and Nile Red) rather than just one (Coumarin 6), we sought to accomplish two goals: first, to broaden the emission spectrum to increase CRI values; second, to have more freedom to create WLEDs with a wider range of SPDs and CCTs. Table 2 lists several WLED configurations along with their respective CRI and CCT values.

Table 2 shows that the CRI values significantly improve (with respect to the monolayer and one-dye luminescent molecular systems), surpassing the value of 80 in nine WLEDs. At the same time, WLEDs of very different CCT are generated. Table 3 shows WLEDs with CRI values above 80, ordered from colder (higher CCT) to warmer (lower CCT).

Table 3 shows we have developed nine WLEDs of high light quality (CRI above 80) with different correlated color temperatures ranging from 2975 K to 6261 K, each of which could efficiently mimic sunlight at a certain time of day. We selected three WLEDs (see Table 4)—corresponding to cold, neutral, and warm WLEDs—to study their optical properties in more detail.

**Figure 3:**
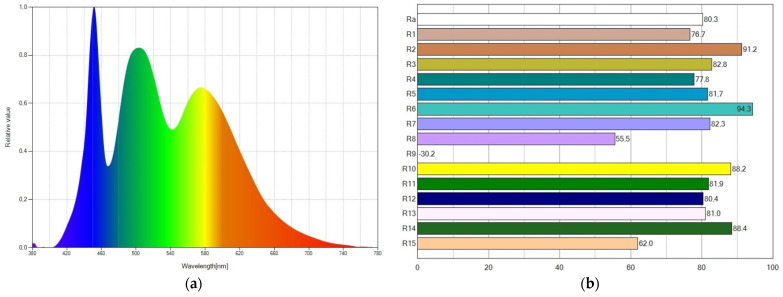
For WLED16, (**a**) spectral power distribution showing a high blue peak and (**b**) CRI values reaching a CRI (R_a_) value of 80.3.

The spectral power distribution and the CRI values of WLED16 are displayed in Figure 3. The wavelengths of emitted light expand to the whole visible spectrum, as depicted in Figure 3a, which accounts for the higher CRI value achieved. Due to the light emissions from the blue LED chip, Coumarin 6, and Nile Red, the SPD exhibits three peaks. The SPD shows a prominent blue peak, resulting in a cool WLED (CCT of 6261 K). The CRI (R_a_) parameter reaches a value of 80.3, as seen in Figure 3b.

**Figure 4:**
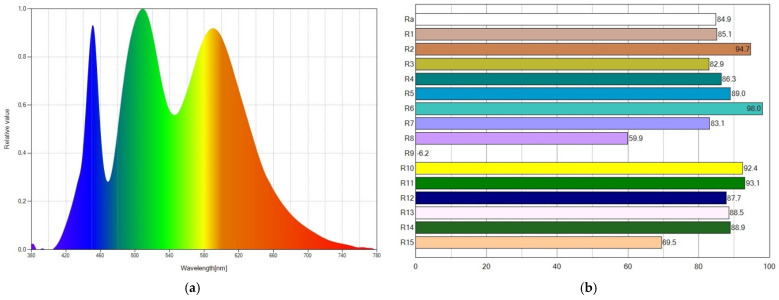
For WLED9, (**a**) spectral power distribution showing a medium blue peak and (**b**) CRI values reaching a CRI (R_a_) value of 84.9.

The spectral power distribution and CRI values of WLED9 are displayed in Figure 4. The wavelengths of emitted light also expand to the whole visible spectrum, as depicted in Figure 4a, which accounts for the high CRI value attained. The SPD also shows three peaks, which we attributed to the light emitted from the blue LED chip, Coumarin6 and Nile Red. A medium blue peak in the SPD results in a neutral WLED (CCT of 4885 K). The CRI (R_a_) parameter reaches a value of 84.9, as seen in Figure 4b.

**Figure 5:**
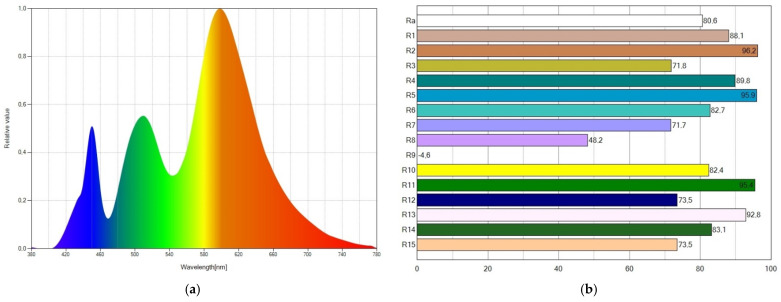
For WLED15, (**a**) spectral power distribution showing a short blue peak and (**b**) CRI values reaching a CRI (R_a_) value of 80.6.

The spectral power distribution and CRI values of WLED15 are displayed in Figure 5. Similar to what occurs in WLED16 and WLED9, the wavelengths of emitted light expand to the whole visible spectrum, as depicted in Figure 5a, which accounts for the high CRI value achieved. The blue LED chip, Coumarin6, and Nile Red are again responsible for the three peaks in the SPD of WLED15. A short blue peak in the SPD results in a warm WLED (CCT of 2975 K). The CRI (R_a_) parameter reaches a value of 80.6, as seen in Figure 5b.

### 2.5. Tunable and Rare-Earth-Free WLEDs

WLEDs with both high CRI and easy tunable CCT values are required; therefore, they are currently attracting considerable research attention. From our results, we concluded that by using only two luminescent species and/or changing their concentration with respect to the polymeric matrix in which they are embedded and/or the thickness of the resulting luminescent layers and/or the relative position of the layers, it is possible to develop WLEDs with a wide range of color temperatures without sacrificing light quality, which is measured through parameters such as CRI. Furthermore, we manufactured these WLEDS by utilizing organic materials, which eliminates the need for rare-earth elements and their associated high risks linked to high costs, monopolies, demand, and geopolitical conflicts.

These WLEDs could be the basic components of a dynamic LED luminaire that uses at least two WLEDs of different color temperatures (for example, a cold and a warm WLED). Thus, by varying the flux of light emitted by each of the individual WLEDs, it is possible to generate light of different color temperatures to recreate the solar cycle and its variations throughout the day, demonstrating the considerable potential of these photoluminescent organic materials in the human-centric lighting sector.

## 3. Materials and Methods

### 3.1. Selection of the Materials

**Figure 6:**
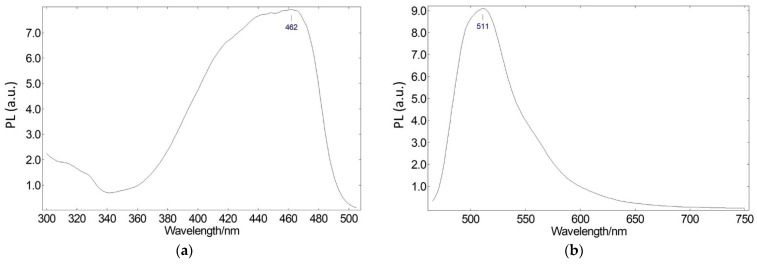
(**a**) Photoluminescence excitation spectrum (peaking at 462 nm) and (**b**) photoluminescence emission spectrum (peaking at 511 nm) of Coumarin 6 green-emitting converter embedded in an EVA polymer.

The selection of the photoluminescent materials was made according to the following criteria. First, we looked for a photoluminescent organic dye whose excitation spectral range overlapped with the emission spectrum of the chosen blue LED chip (see Figure 1). A molecule that satisfies this criterion is Coumarin 6. The excitation and emission spectra of Coumarin 6 dye embedded in EVA polymer are displayed in Figure 6. The visible excitation spectrum, as shown in Figure 6a, ranges from the ultraviolet to 500 nm, with an excitation peak at 462 nm, which is quite near to the blue LED’s emission peak. The blue LED + phosphor system’s emission range, as shown in Figure 6b, is extended compared with that of a pure blue LED chip, peaking at 511 nm.

**Figure 7:**
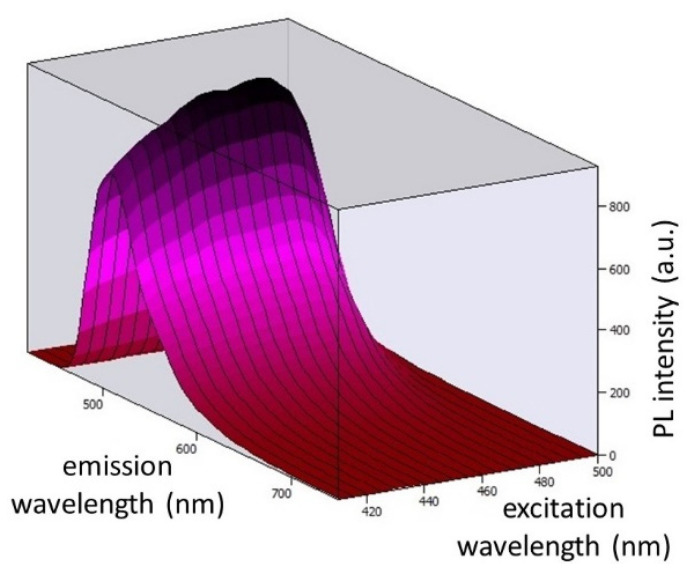
Three-dimensional photoluminescence spectra of Coumarin 6 dye embedded in EVA polymer at excitation wavelengths ranging from 410 nm to 500 nm and emission wavelengths ranging from 450 nm to 750 nm.

Figure 7 displays the 3D photoluminescent (PL) spectra of Coumarin 6 dye embedded in EVA polymer at excitation wavelengths ranging from 410 nm to 500 nm (corresponding to the spectrum of light emitted by the blue LED chip). By observing the spectra, we concluded that our chosen blue LED chip can excite Coumarin 6 embedded in EVA.

**Figure 8:**
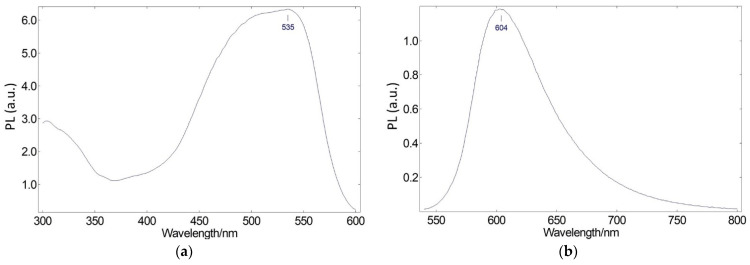
(**a**) Photoluminescence excitation spectrum (peaking at 535 nm) and (**b**) photoluminescence emission spectrum (peaking at 604 nm) of Nile Red red-emitting converter embedded in an EVA polymer.

Then, to further broaden the spectral range of the emitted light, we searched for a second photoluminescent organic dye. Nile Red has a wide excitation spectrum that may partially harness both the light released by the blue LED chip and the Coumarin 6 dye. The excitation and emission spectra of Nile Red dye embedded in EVA polymer are displayed in Figure 8. The visible excitation spectrum, as shown in Figure 8a, ranges from the ultraviolet to 600 nm, peaking at 535 nm and considerably overlapping with Coumarin 6′s emission spectrum. Regarding the emission spectrum (Figure 8b), there is a peak at 604 nm, enabling the generated light’s spectral range to be expanded even further.

**Figure 9:**
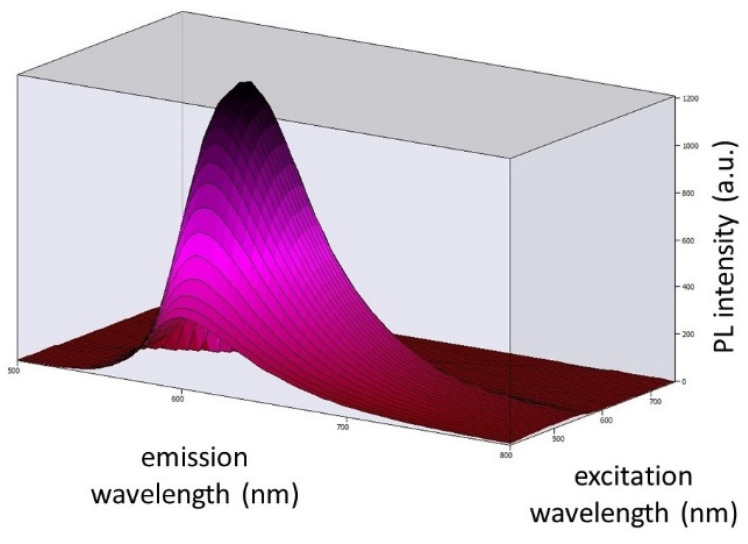
Three-dimensional photoluminescence spectra of Nile Red dye embedded in EVA polymer at excitation wavelengths ranging from 410 nm to 740 nm and emission wavelengths ranging from 500 nm to 800 nm.

Figure 9 shows the 3D photoluminescent (PL) spectra of Nile Red dye embedded in EVA polymer at excitation wavelengths ranging from 410 nm to 740 nm (corresponding to the light spectra emitted by the blue LED chip and Coumarin 6 dye). By observing the spectra, we concluded that the blue LED chip + Coumarin 6 molecular system can excite Nile Red embedded in EVA.

### 3.2. Experimental Details and Methods

First, 25 g EVA were added to 200 mL dichloromethane and stirred at 60 °C until complete dissolution was achieved. Several solutions of this type were prepared. Taking 25 g EVA as a reference, different amounts of an organic dye (Coumarin 6 or Nile Red) were added to each solution, thus obtaining solutions with different concentrations of dye (expressed as weight percentages with respect to EVA).

Then the solvent was evaporated, and photoluminescent layers of dye-doped EVA polymer were formed. These layers were taken to a hot plate press, where temperature and pressure were applied to achieve uniformity of the layers as well as the required thickness.

## 4. Conclusions

There has been a revolution in the lighting sector, with tungsten sources being replaced by more energy-efficient and longer-life LED bulbs. In addition, white light-emitting diode (WLED) technology has further advantages, such as a greater degree of spectral power distribution (SPD) and correlated color temperature (CCT) adjustment, which is required for human-centric lighting. Human-centric lighting preserves circadian rhythms and avoids chronodisruption by dimming and changing the SPD and CCT values of a smart light bulb to appropriately mimic the spectrum and irradiance levels of sunlight throughout the day.

Lighting applications make use of rare-earth elements (REEs). They are frequently used as phosphors (wavelength converters) in WLEDs. However, manufacturers are concerned about supply limits, including growing costs, and China’s monopoly over the supply of the vast majority of REEs. Consequently, the search for alternatives to REEs is a hot topic of research and interest.

In our study, we developed several rare-earth-free WLEDs of different color temperatures using the same blue LED chip (with an emission peak at 450 nm), two photoluminescent organic dyes (Coumarin 6 and Nile Red), and ethylene vinyl acetate (EVA) copolymer as the host material. By simply changing the concentrations of the photoluminescent dyes (with respect to the EVA polymeric matrix) and/or by varying the thicknesses and/or relative positions of the photoluminescent layers, WLEDs of different CCT (ranging from 2975 K to 6261 K) and high chromatic reproduction index (CRI) values (superior to 80) can be manufactured. These WLEDs could provide the basic components of a dynamic luminaire (based on at least two WLEDs of different color temperatures and variable intensities), which can recreate the solar cycle and its variations throughout the day. Our results demonstrate for the first time ever the potential of using organic materials instead of rare-earth elements in the field of human-centric lighting.

## Figures and Tables

**Table 1:** A summary of WLED devices (1, 2, and 3) along with their optical properties.

WLED	% Dye (Layer 1)	Thickness (Layer 1)	CRI	CCT (K)
WLED1	C6 0.0100%	0.5 mm	55.6	34,565
WLED2	C6 0.0175%	0.5 mm	36.7	8708
WLED3	C6 0.0250%	0.2 mm	43.2	10,046

**Table 2:** A summary of WLEDs devices (4 to 21) along with their optical properties.

WLED	% Dye (Layer 1)	Thickness (Layer 1)	% Dye (Layer 2)	Thickness (Layer 2)	CRI	CCT (K)
WLED4	C6 0.025%	0.2 mm	NRed 0.010%	0.2 mm	74.1	6892
WLED5	NRed 0.010%	0.2 mm	C6 0.025%	0.2 mm	70.7	6870
WLED6	C6 0.025%	0.2 mm	NRed 0.010%	0.5 mm	81.2	4886
WLED7	NRed 0.010%	0.5 mm	C6 0.025%	0.2 mm	81.1	5924
WLED8	C6 0.025%	0.2 mm	NRed 0.010%	0.8 mm	66.4	3077
WLED9	NRed 0.010%	0.8 mm	C6 0.025%	0.2 mm	84.9	4885
WLED10	C6 0.025%	0.2 mm	NRed 0.020%	0.2 mm	82.7	5665
WLED11	NRed 0.020%	0.2 mm	C6 0.025%	0.2 mm	78.5	6207
WLED12	C6 0.025%	0.2 mm	NRed 0.020%	0.5 mm	44.6	2060
WLED13	NRed 0.020%	0.5 mm	C6 0.025%	0.2 mm	86.2	3712
WLED14	C6 0.025%	0.2 mm	NRed 0.020%	0.8 mm	32.6	1747
WLED15	NRed 0.020%	0.8 mm	C6 0.025%	0.2 mm	80.6	2975
WLED16	C6 0.025%	0.2 mm	NRed 0.005%	0.2 mm	80.3	6261
WLED17	NRed 0.005%	0.2 mm	C6 0.025%	0.2 mm	73.8	6650
WLED18	C6 0.025%	0.2 mm	NRed 0.005%	0.5 mm	83.0	5429
WLED19	NRed 0.005%	0.5 mm	C6 0.025%	0.2 mm	79.1	6266
WLED20	C6 0.025%	0.2 mm	NRed 0.005%	0.8 mm	78.5	4260
WLED21	NRed 0.005%	0.8 mm	C6 0.025%	0.2 mm	82.6	5312

**Table 3:** A summary of best WLED devices (listed from higher CCT to lower CCT) along with their optical properties.

WLED	% Dye (Layer 1)	Thickness (Layer 1)	% Dye (Layer 2)	Thickness (Layer 2)	CRI	CCT (K)
WLED16	C6 0.025%	0.2 mm	NRed 0.005%	0.2 mm	80.3	6261
WLED7	NRed 0.010%	0.5 mm	C6 0.025%	0.2 mm	81.1	5924
WLED10	C6 0.025%	0.2 mm	NRed 0.020%	0.2 mm	82.7	5665
WLED18	C6 0.025%	0.2 mm	NRed 0.005%	0.5 mm	83.0	5429
WLED21	NRed 0.005%	0.8 mm	C6 0.025%	0.2 mm	82.6	5312
WLED6	C6 0.025%	0.2 mm	NRed 0.010%	0.5 mm	81.2	4886
WLED9	NRed 0.010%	0.8 mm	C6 0.025%	0.2 mm	84.9	4885
WLED13	NRed 0.020%	0.5 mm	C6 0.025%	0.2 mm	86.2	3712
WLED15	NRed 0.020%	0.8 mm	C6 0.025%	0.2 mm	80.6	2975

**Table 4:** Description of three selected WLED devices (16, 9, and 15) along with their optical properties.

WLED	% Dye (Layer 1)	Thickness (Layer 1)	% Dye (Layer 2)	Thickness (Layer 2)	CRI	CCT (K)
WLED16	C6 0.025%	0.2 mm	NRed 0.005%	0.2 mm	80.3	6261
WLED9	NRed 0.010%	0.8 mm	C6 0.025%	0.2 mm	84.9	4885
WLED15	NRed 0.020%	0.8 mm	C6 0.025%	0.2 mm	80.6	2975

## Data Availability

The data supporting the findings of this study are available within the article.
